# Acclimation during Embryogenesis Remodulates Telomerase Activity and Gene Expression in Baikal Whitefish Larvae, Mitigating the Effects of Acute Temperature Stress

**DOI:** 10.3390/ani14192839

**Published:** 2024-10-02

**Authors:** Anastasiya G. Koroleva, Eugenia A. Vakhteeva, Alexander A. Epifantsev, Lyubov V. Sukhanova, Vera M. Yakhnenko, Olga Yu. Glyzina, Lyubov I. Tolstikova, Valeria M. Cherezova, Tuyana V. Sidorova, Sergey A. Potapov, Sergey V. Kirilchik, Yulia P. Sapozhnikova

**Affiliations:** Limnological Institute Siberian Branch of the Russian Academy of Sciences, 3 Ulan-Batorskaya, Irkutsk 664033, Russialsukhanova@yandex.ru (L.V.S.);

**Keywords:** telomere, telomerase, antioxidant enzymes, Q-PCR, transcriptome, Baikal whitefish, acclimation, temperature stress

## Abstract

**Simple Summary:**

Temperature acclimation enables animals, especially aquatic ones, to safely survive climate fluctuations in the natural environment. The aim of our work was to study how temperature acclimation in aquaculture affects cold-water Baikal whitefish at the embryonic stage and their well-being during a heat shock (24 °C). Selected molecular markers (telomere length, telomerase activity, and expression of target genes) showed that acclimation at the early developmental stages has a positive effect on the Baikal whitefish larvae and allows them to tolerate acute temperature stress without the harmful consequences. The data obtained will improve the survival of fish and increase their plasticity under aquaculture conditions.

**Abstract:**

Acclimation through the hormesis effect increases the plasticity of organisms, which has been shown for many ectothermic animals, including fish. We investigated the effect of temperature acclimation in Baikal whitefish *Coregonus baicalensis* (Dybowski, 1874). Telomere length, telomerase activity, and the expression of genes, whose products are involved in the regulation of telomere length and defense against reactive oxygen species, were selected to assess the state of the larvae. Acclimation and acute temperature stress (+12 °C) had no effect on telomere length, but altered telomerase activity (acclimation decreased it; stress increased it) and the levels of genes expression. Under stress, the expression of superoxide dismutase genes was increased in acclimated larvae and that of glutathione peroxidases in non-acclimated larvae, which may indicate lower reactive oxygen species formation and slower antioxidant responses in acclimated fish. The expression of some telomere-related genes was reduced under temperature stress, but the expression of the *tzap* and *smg* genes, whose products improve the control of telomere length by preventing them from lengthening or shortening, was increased in acclimated individuals. The data obtained indicate a positive effect of acclimation on the state of the Baikal whitefish larvae by remodulation of their telomerase activity and the transcriptional profile.

## 1. Introduction

The natural variability of the environment influences the state of organisms to different degrees. For example, insignificant variations in temperature, light, and water chemistry do not significantly change the physiology of hydrobionts due to their phenotypic plasticity [1]. However, deviations in habitat conditions occur, triggering allostasis, where stability is achieved through significant changes in physiology [2,3]. While the first variant can be considered as acclimatization (when several abiotic factors change) or acclimation (when one factor changes) without major negative effects on the organism, the second variant leads to distress and many harmful consequences. The physiological response to acclimation and stress in animals is regulated by the hypothalamic–pituitary–adrenal axis (in fish, the hypothalamic–pituitary–interrenal axis, HPIA) by catecholamines, glucocorticoids, and other hormones [4,5,6,7,8]. However, unfavorable effects depend on the intensity and timing of exposure to the stressor and are mitigated in the case of acclimation, as it is carried out gradually, within the reaction norm of a particular species, and prepares the organism for non-standard conditions. Acclimation has acute (initial reaction to the change) and chronic (repeated reaction) phases, which, however, are always gentler than distress and do not lead to the death of the organism [7].

The most important abiotic factor in the ecology of fish and other ectothermic hydrobionts is temperature, on which enzymatic reactions, cellular respiration, oxygen consumption, and, consequently, metabolic rate and the fulfillment of vital processes such as digestion, movement, growth, and reproduction depend [8,9]. An increase in temperature enhances oxygen consumption and the production of reactive oxygen species (ROS), which often leads to oxidative stress and a higher activity of antioxidant enzymes in fish [10,11,12,13]. Under severe and prolonged stress, the cell’s defense systems cannot cope with the load and apoptosis occurs [14]. However, in some animals acclimated to a certain temperature, a different pattern is observed due to the hormesis effect, although the response to acclimation is species-specific [15,16].

Many components of the cell, including telomeres at the end of chromosomes, are damaged in the pathway from increased ROS to apoptosis [17,18]. Telomere length serves as a marker of an organism’s well-being [19,20,21] and is regulated by telomerase and other proteins [22,23]. Temperature changes significantly impact telomere length and telomerase activity in many fish species [24,25,26,27,28], and also alter gene expression [29,30]. This allows us to use telomere biology and the activity of target genes, such as those encoding antioxidant enzymes, during acclimation and under stress as indicators of the state of the organism. To examine these temperature effects, we chose the cold-water Baikal whitefish *Coregonus baicalensis* (Dybowski, 1874). We measured telomere length (TL), telomerase activity (TA) and the expression of two groups of genes: (1) those involved in the regulation of TA and TL and (2) those acting as the first line of defense against ROS.

Whitefish are cold-water benthic fish, with juveniles growing optimally at water temperatures between 13 and 18 °C [31,32,33,34], but temperatures above 26 °C are lethal for some species [35,36,37]. In aquaculture, temperature fluctuations of 6–9 °C regularly occur when cooling systems fail or during fish transportation, which has a negative impact on fish health and survival rates [35,38]. However, acclimation can mitigate the effects of acute stress, as previously demonstrated in *C. clupeaformis* [39]. The early stages of embryogenesis of *C. baicalensis* are very sensitive to temperature; therefore, acclimation, or pre-adaptation, in whitefish should occur after the main organs, including the circulatory system, have formed, specifically on the 45th day after fertilization [40]. It is known that in many fish farms, whitefish are released into rivers at the larval stage, where they exhibit high flexibility [41,42]. This approach is cost-effective. However, after hatching and before release into the natural environment, larvae need time to switch to an external feeding (day 8), develop fins (day 20), and fill their swim bladders with air (day 30) [40]. When such non-acclimated larvae are released into rivers, they may still experience stress.

Our experiment was designed to correspond to the developmental characteristics of whitefish. The embryos were acclimated to the upper limit of their physiological norm at 9 °C [39,40,43,44]. After hatching, the larvae were reared at a physiologically normal temperature of 12 °C for their age [40,44]. Acute stress was induced at a sublethal temperature 24 °C [44] when the larvae reached 1 month of age to evaluate the stress response of both acclimated and non-acclimated individuals. The results of this study will enhance our understanding of larval health and improve conditions for whitefish in aquaculture, ultimately reducing their stress upon release.

## 2. Materials and Methods

### 2.1. Rearing of the Baikal Whitefish Larvae and Experimental Setup

The eggs/embryos of native Baikal whitefish were collected and fertilized in Chivyrkuisky Bay on Lake Baikal, in the spawning grounds of whitefish (53°42′14.3″ N 109°02′16.8″ E). Only the Chivyrkuisky whitefish occur in this region and form a separate population [45]. All manipulations related to artificial fertilization were performed in accordance with [40].

The Baikal whitefish embryos were placed in Weiss experimental and control incubators on 30 December 2022. Heating/acclimation of the embryos began in the experimental incubators on 16 February 2023. These incubators were converted to a closed cycle with a supply aquarium that featured active forced aeration by a compressor and a three-stage water heater that increased the temperature from an initial 3–3.5 °C to 9 °C. The physiological temperature norm for the genus *Coregonus* at this age is 5–11 °C [39,40,43], while for *C. baicalensis* it is 7–14 °C [40,44]. Heating was repeated every three days in the following these steps: 1. gradual heating to 9 °C over one hour; 2. maintaining the temperature at 9 °C for one hour; 3. gradual cooling back to the initial temperature over one hour. This heating procedure was repeated until hatching was completed on 21 April 2023. The mortality of the heated embryos was 15% higher than in the control group.

After hatching, both control and acclimated larvae were kept under the same optimal aquaculture conditions at 12 °C [40,44] in the “Experimental Freshwater Aquarium Complex for Baikal Hydrobionts” at LIN SB RAS for 1 month.

**Figure 1:**
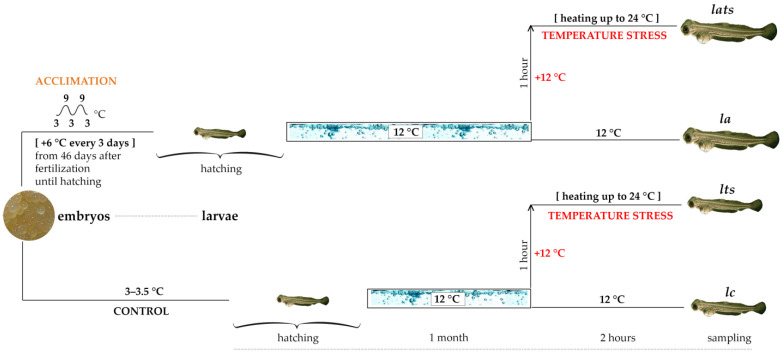
Scheme of the experiment. Some of the whitefish embryos were acclimated to temperature differences from 3 to 9 °C (***la***), and some of the embryos were incubated under normal conditions (3–3.5 °C) (***lc***). After hatching, the larvae were kept at 12 °C for one month; two groups (***lts*** and ***lats***) were exposed to temperature stress by heating to 24 °C (+12 °C) and two groups (***lc*** and ***la***) were left as the control groups. Material was taken from all four groups immediately after the stress. ***Lc***—unexposed control individuals; ***lts***—unexposed individuals under temperature stress; ***la***—acclimated individuals at the embryo stage; ***lats***—acclimated individuals under temperature stress.

A total of 120 larvae were used for the experiment. They were divided into four groups (30 individuals per group): ***lc***—unexposed control individuals (larvae); ***la***—acclimated individuals at the embryo stage; ***lts***—unexposed individuals under temperature stress; and ***lats***—acclimated individuals under temperature stress. Temperature stress modeling consisted of heating the water to 24 °C (+12 °C) for one hour, maintaining this temperature for the following two hours, and slowly cooling for five hours (***lts*** and ***lats*** groups). Sampling was performed in both groups immediately after two hours of stress conditions. The samples of the ***la*** and ***lc*** groups were taken at the same time. The scheme of the experiment is shown in Figure 1.

Larvae were taken from each group for three analyses (10 individuals per analysis): they were fixed whole in 96% alcohol (for genomic DNA isolation) and in TRIzol reagent (Thermo Fisher Scientific, Waltham, MA, USA) (for RNA isolation), and frozen in liquid nitrogen (for protein isolation). To ensure the purity of DNA and protein isolation and the analysis of these components in different tissues, larvae were separated by tissues and organs (gills, internal organs, and muscles), from which DNA and proteins were isolated separately. They were then further analyzed. Because there were no significant differences in patterns of telomere length and telomerase activity between tissues, data were pooled into an average for each individual. RNA was isolated directly from the whole larvae.

### 2.2. Sequencing and Transcriptome Analysis

RNA was isolated using the TRIzol reagent according to the manufacturer’s recommendations. RNA concentration was measured using an EzDrop1000 spectrophotometer (BLUE-RAY BIOTECH, Taiwan). Further, 5–8 samples from each group (***lc***, ***la***, ***lts***, and ***lats***) were combined into a mixture to obtain four RNA pools with a concentration of 220–1000 ng per μL. The pools had the same names: ***lc***, ***la***, ***lts***, and ***lats***. mRNA was separated from total RNA using magnetic beads with poly-T primers. After fragmentation, the first-strand cDNA was synthesized using random hexamer primers, followed by second-strand cDNA synthesis using dTTP for the non-directional library. It was ready after end repair, A-tailing, adapter ligation, size selection, amplification, and purification. Libraries were checked with Qubit and real-time PCR for quantification and a bioanalyzer for size distribution detection. Quantified libraries were pooled in equimolar amounts and sequenced on an Illumina NovaSeq 6000 high-throughput sequencer using a NovaSeq 6000 Reagent Kit v1.5 in 150 bp paired-end reads from “Novogene” company (Beijing, China).

Our raw data in fastq format were first processed through in-house perl scripts. In this step, clean data were obtained by removing reads containing adapter, reads containing ploy-N, and low-quality reads from raw data. At the same time, Q20, Q30, and GC contents were calculated. All the downstream analyses were based on the clean data with high quality. Paired-end clean reads were aligned to the reference genome of the Atlantic salmon *Salmo salar* (https://www.ncbi.nlm.nih.gov/datasets/genome/GCF-905237065.1/, accessed on 30 September 2024) using Hisat2 v2.0.5 [46]. The mapped reads of each sample were assembled by StringTie v1.3.3b in a reference-based approach. StringTie uses a novel network flow algorithm as well as an optional de novo assembly step to assemble and quantitate full-length transcripts representing multiple splice variants for each gene locus [47]. FeatureCounts v1.5.0-p3 was used to count the read numbers mapped to each gene. Then, the FPKM of each gene was calculated based on the length of the gene and the read count mapped to this gene [48]. FPKM, expected number of fragments per kilobase of transcript sequence per millions base pairs sequenced, considers the effect of sequencing depth and gene length for the read count at the same time, and is currently the most commonly used method for estimating gene expression levels. Prior to differential gene expression analysis, for each sequenced library, the read counts were adjusted by the edgeR program package through one scaling normalized factor. Differential expression analysis of two conditions was performed using the edgeR R package 3.22.5 [49]. The P values were adjusted using the Benjamini and Hochberg method. A corrected P value of 0.05 and absolute foldchange of 2 were set as the threshold for significantly differential expression. Gene Ontology enrichment analysis of differentially expressed genes and an analysis of the KEGG pathways of them were implemented by the clusterProfiler R package, in which gene length bias was corrected [50,51]. GATK v4.1.1.0 software was used to perform SNP calling. Raw vcf files were filtered with the GATK standard filter method together with other parameters (cluster: 3; window size: 35; QD < 2.0; FS > 30.0; DP < 10) [52]. rMATS 4.1.0 software was used to analyze the alternative splicing event [53]. Protein–Protein Interaction analysis (PPI) of differentially expressed genes was based on the STRING database, which was known and predicted.

This work focuses on the expression of genes involved in the regulation of telomerase activity, telomere maintenance, and defense against ROS. Among the annotated sequences, we found genes encoding TERT (the major protein component of telomerase), other components of the telomerase and Shelterin complexes, and transcripts of genes encoding enzymes of the first line of defense against oxidative stress: various forms of superoxide dismutase and glutathione peroxidase, catalase, and glutathione S-transferase (Table 1). Genes with parameter values |log2FoldChange| ≥ 1 and P adjusted < 0.05 (P adj) were considered as differentially expressing genes. The program R studio v. 4.3.3 was used to generate heat maps of the expression level of the target genes using FPKM and P adj.

### 2.3. Determination of the Telomere Length and Telomerase Activity

DNA was isolated using the phenol/chloroform method [54,55]. The relative TL values (telomere DNA amount/the reference single-copy gene amount, T/S) were determined by quantitative PCR (Q-PCR) using the Cawthon method with standard curves for telomeric sequences and the reference gene [56]. The primers Tel1 and Tel2 were used for telomeric sequences [56]. A recombination-activated gene was chosen as the reference gene (*rag1*). The primers were selected from the known sequence of this gene for *Coregonus clupeaformis* (XM_041838002.2) and had the following structure: RAG1_F 5′-CTTCAAAGTGGACGTGACGG-3′ and RAG1_R 5′-CCAGGCTCTCTTCTCACCAA-3′. The Primer-BLAST program was used (https://www.ncbi.nlm.nih.gov/tools/primer-blast/index.cgi?GROUP_TARGET=on, accessed on 30 September 2024). The amplicon length was 293 bp. The reaction mixture for the reference gene contained normal buffer, 2.5 mM magnesium chloride, 0.25 mM of each dNTP, 0.2 units of Snp polymerase (ZAO Evrogen, Moscow, Russia), 0.5-fold SYBR Green (ZAO Evrogen, Moscow, Russia), 0.2–0.3 ng of DNA, and 0.5 pmol of each primer. In the reaction with telomeric primers, 0.17 pmol of Tel1 and 0.5 pmol of Tel2 were added to the same mixture instead of the primers for the reference gene. The reference gene was amplified under touch-down conditions. First, the Snp polymerase was activated at 95 °C for three minutes. Then, the cycle was repeated 7 times with a gradual decrease in the annealing temperature by 1 °C: 95 °C for 10 s, 69–63 °C for 15 s, and 72 °C for 15 s. For the next 40 cycles, the annealing temperature was 63 °C, while the other conditions were maintained. Telomeric repeats were amplified in a cycle of 95 °C for 15 s, 54 °C for 2 min (repeated 45 times). All standard curves had a coefficient of determination close to 1 (for gene *rag1* R^2^ = 0.995 ± 0.0006, for telomeres R^2^ = 0.997 ± 0). The efficiency of the reactions for telomeric repeats was 73.4 ± 3.2%, and for the reference gene it was 90.3 ± 12.2%. There were three replicates for each sample, and the test itself was repeated twice. Q-PCR was performed using a CFX instrument (BioRad, Hercules, CA, USA). The T/S values of the different tissues were combined and presented as mean ± standard deviation (mean ± SD) for comparison with the gene expression data.

Total protein was isolated using CHAPS buffer as described in [57]. Telomerase activity was quantitatively determined using the Q-TRAP method on a Rotor-Gene Q 6000 instrument (“QIAGEN”, Hilden, Germany) according to [57,58]. Protein concentration was measured using the Bradford method [59]. The relative TA was calculated using the ΔΔCt method [60], which was implemented in Rotor-Gene software v. 2.3.1. The TA of the first control fish was set as 1, and the remaining samples were calculated relative to the selected calibrator [57]. The TA values of the different tissues were also combined and presented as mean ± standard deviation (mean ± SD).

### 2.4. Statistical Analysis

As the number of individuals was limited (10 individuals in each group), the non-parametric Kruskal–Wallis test was used to compare the TL and TA values in the control, acclimated, and temperature-exposed individuals (K–W test). The zero hypothesis of no response to temperature and acclimation was refuted if the significance value was less than 0.05. Analysis of variance (ANOVA) was used for biometric comparisons. Calculations and visualization were performed in Statistica 10.

## 3. Results

**Figure 2:**
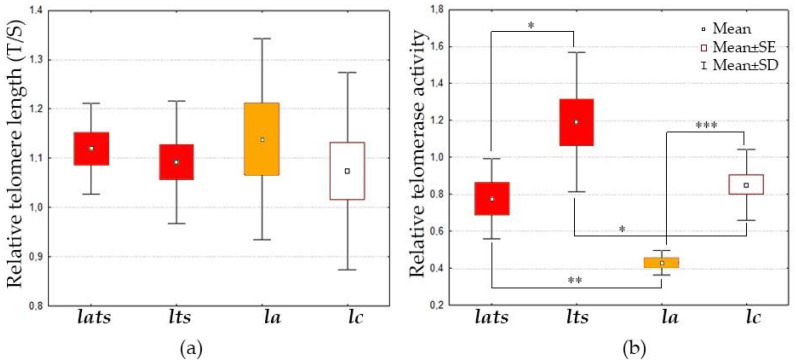
Relative telomere length (**a**) and relative telomerase activity (**b**) in the Baikal whitefish larvae. Groups of larvae: ***lc***—unexposed control individuals; ***lts***—unexposed individuals under temperature stress; ***la***—acclimated individuals at the embryo stage individuals; ***lats***—acclimated individuals under temperature stress. Each group contains 6–15 individuals. The red color indicates groups that were exposed to acute temperature stress. The orange color indicates only acclimated groups. Asterisks indicate reliable differences between groups (Kruskal–Wallis test, * < 0.05, ** < 0.01, *** < 0.001); SE—the standard error of the mean; SD—the standard deviation from the mean.

During the experiment, it was observed that acclimated individuals were larger than non-acclimated ones. The mass and size of acclimated larvae were significantly larger (0.05 ± 0.01 g and 23.1 ± 1.4 mm) than those of non-acclimated larvae (0.01 ± 0.003 g and 14.7 ± 2.1 mm) (n = 45, ANOVA, P < 0.05). Large and small individuals did not differ significantly in TL, nor did acclimated (***la***), stressed (***lats***, ***lts***), and control individuals (***lc***) (Figure 2a). However, acclimation had a significant effect on TA, which was reduced in the ***lats*** group compared to controls (K–W test: H (1, N = 21) =13.4, P = 0.0003; Figure 2b). Acute temperature stress led to increased TA in acclimated ***lats*** individuals (K–W test: H (1, N = 13) = 8.2, P = 0.0043) and non-acclimated ***lts*** individuals (K-W test: H (1, N = 23) = 4.6, P = 0.0322; Figure 2b). The latter showed higher TA under acute stress than acclimated ***lats*** (K–W test: H (1, N = 15) =4, P = 0.0451, Figure 2b).

**Figure 3:**
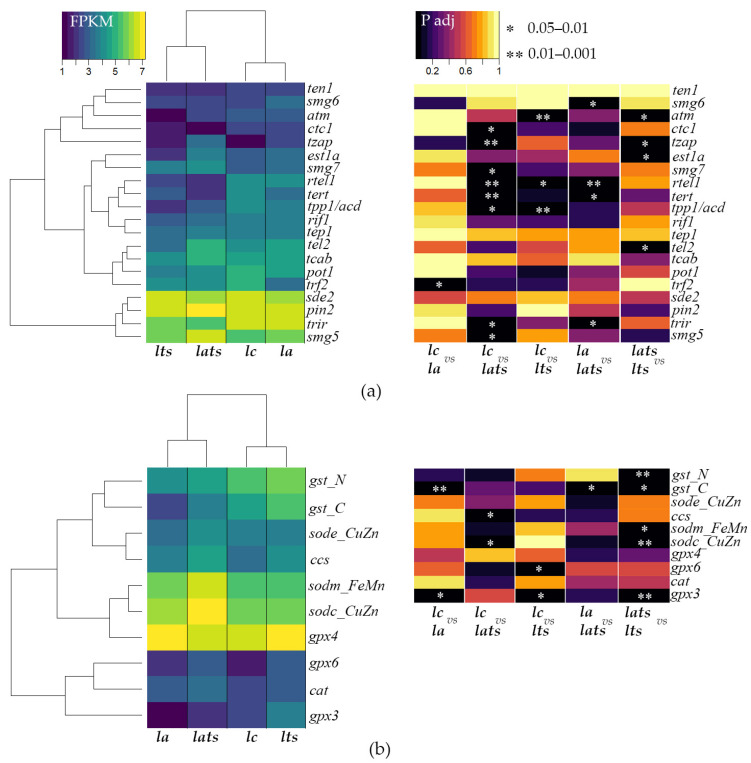
Heat maps of gene expression generated by FPKM (normalized data) and P adj confidence values. (**a**): Expression levels of genes encoding proteins involved in the regulation of TL and TA. (**b**): Expression levels of genes encoding antioxidant enzymes. * and ** indicate significant differences between gene expression in the compared groups (P adj < 0.05; log2FoldChange ≥ 1). Groups: ***lc***—unexposed control individuals; ***lts***—unexposed individuals under temperature stress; ***la***—acclimated at the embryo stage individuals; ***lats***—acclimated individuals under temperature stress.

The accession number for uploading transcriptome data is _________ in GEO-NCBI database (link will be available soon). These data are analyzed and discussed in [61]. In the present work, we analyzed genes involved in telomere length regulation and encoding antioxidant enzymes. The FPKM and P adj values indicate a significant difference in the expression of target genes in acclimated and control individuals, as well as in individuals subjected to acute stress (Figure 3). Acclimation had almost no effect on the expression of most genes involved in the regulation of TL; only the expression of the *trf2* gene was downregulated (Figure 3a). The acclimation decreased the activity of the *gpx3* and *gst* genes without affecting the expression of other genes encoding enzymes involved in protection against ROS (Figure 3b). Acute temperature stress in acclimated individuals led to a significant decrease in the activity of genes involved in the maintenance of telomere regions *ctc1*, *rtel1*, *tert*, *tpp1*, and *trir* (compared to control group ***lc***) and in addition to these genes *smg6* (compared to group ***la*** with acclimated individuals) (Figure 3a). At the same time, the expression of *tzap*, *smg7*, *smg5*, *sodc_CuZn*, *ccs*, and *gst* was increased, but only in comparison to ***la*** (Figure 3a,b). Acute stress in non-acclimated fish also resulted in decreased activity of several other genes involved in TL maintenance (*tpp1*/*acd*, *rtel1*, *atm*) and increased activity of glutathione peroxidase genes (*gpx3* and *gpx6*) (Figure 3a,b). When we compare the gene expression profiles of acclimated and non-acclimated individuals under conditions of acute temperature stress with each other rather than with control individuals, we find significant differences: *est1a*, *tel2*, *atm*, and *tzap*, as well as the superoxide dismutase genes (*sodc_CuZn* and *sodm_FeMn*) are reduced in the ***lts*** group, while the expression of glutathione peroxidase and glutathione-S-transferase genes is upregulated compared to the ***lats*** group (Figure 3a,b).

## 4. Discussion

### 4.1. Stability of Telomere Length Is Ensured by Different Telomerase Activity in Acclimated and Non-Acclimated Individuals under Acute Temperature Stress

Acclimation is considered an effective means of making an organism more resistant to stress [62,63]. It influences cellular processes and physiology by having a hormetic effect. Telomere biology may also help adapt to environmental changes. The telo-hormesis hypothesis states that changes in TL and TA are adaptive and help organisms survive unfavorable conditions [64].

**Figure 4:**
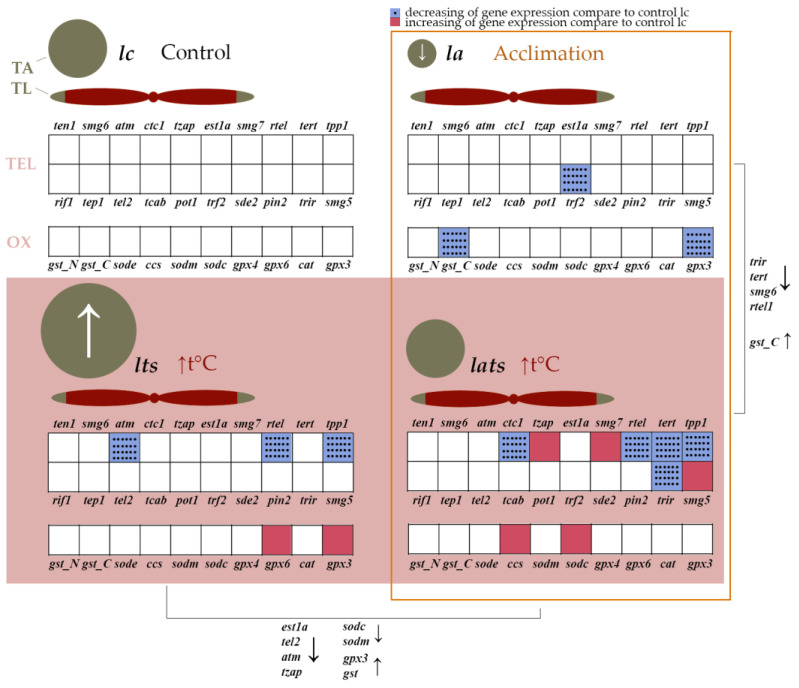
Generalized scheme of the results on telomerase activity (TA), telomere length (TL), and gene expression (TEL—involved in the regulation of telomere length; OX—genes of enzymes protecting against ROS). The size of the circle corresponds to the relative numerical value of TA. Arrows indicate that the parameters are increasing or decreasing. Acclimated groups are highlighted with an orange box; groups exposed to acute temperature stress are highlighted with a pink rectangle and marked with ↑t°C. All groups were compared with the control group, i.e., changes in TA and gene expression are shown relative to ***lc***. Changes in gene activity when comparing the acclimated groups of ***la*** and ***lats*** with each other are shown on the right: change relative to ***la***. Changes in gene activity between the groups exposed to acute temperature stress, ***lts*** and ***lats***, are shown from bottom to top: change relative to ***lats***. Groups: ***lc***—unexposed control individuals; ***lts***—unexposed individuals under temperature stress; ***la***—acclimated individuals at the embryo stage; ***lats***—acclimated individuals under temperature stress.

Acclimation at the embryonic stage has no effect on the TL of Baikal whitefish larvae (Figure 2a), but it significantly reduces TA (Figure 2b and Figure 4). Since TA was analyzed one month after acclimation, the results obtained may be explained by the remodeling of enzyme function to protect the cell from the effects of temperature stress, including increased ROS production. TA changes differently in fish when they are exposed to various stressors: starvation, for example, reduces TA, while hypoxia has no effect on TA [65]. To our knowledge, there are no studies that have investigated the effects of acclimation on telomere biology. The data obtained in this work are the first to confirm the positive effect of acclimation on TL regulation due to changes in TA, especially its decrease. It is noteworthy that the expression of the *tert* gene remains stable at reduced TA levels in acclimated individuals, which is most likely related to the formation of adaptation to temperature fluctuations (Figure 4). There are examples in the literature of both a positive correlation between TA and *tert* gene expression [65,66], as well as examples of their independent regulation [67]. The latter scenario may be explained by non-canonical functions of telomerase, in which *tert* transcripts are involved in other processes (e.g., regulation of proliferation, apoptosis, and autophagy) [68,69]. It is possible that the non-canonical functions of telomerase do not change during acclimation in Baikal whitefish, and that *tert* expression remains at the same level, which may also indicate a normal state of the organism.

We observed another interesting effect of acclimation. In our experiment, the acclimated Baikal whitefish larvae were significantly larger than the control larvae. There are two possible reasons for this: the earlier hatching and the direct effect of temperature on their growth. In the biology of telomeres in mammals, body size plays in important role [70,71,72,73], but in ectothermic vertebrates, the regulation of TL and TA depends more on other factors: the evolutionary history of the species, the conditions of its habitat, and species-specific growth patterns [74]. As ectothermic animals, fish are particularly sensitive to environmental changes [75,76] and have considerable adaptive potential [77,78]. In our case, TL did not change, but TA decreased in the larger acclimated larvae (Figure 2). In other fish, different types of relationships between telomeres and body size were found. In brown trout (*Salmo trutta*), for example, larger individuals living in warmer water had shorter telomeres than smaller individuals of the same age [26]. In another experiment involving starvation and compensatory growth in the same species, no differences in TL were observed during the experiment, but a negative correlation was found between the rate of TL change and the initial size of the fish [79]. In Atlantic salmon (*Salmo salar*), *tert* transcripts (which are often correlated with TA) were present, but the expression level of this gene did not change during growth or under unfavorable conditions, while TL decreased [80]. In rainbow trout (*Oncorhynchus mykiss*), a comparison of TL between normal and dwarf individuals showed no differences [81]. In other non-salmonid fish, the increase in size and growth correlated positively with TA [65,82]. All these data suggest that the relationship between growth, size, and telomere biology is species-specific. The observed changes in body size and TA levels in acclimated Baikal whitefish could be a result of hormesis.

Acute temperature stress as well as acclimation did not lead to changes in TL in either acclimated or non-acclimated *C. baicalensis* individuals. However, TA increased in the non-acclimated individuals, while acclimated larvae showed no changes in TA (Figure 2 and Figure 4). This suggests the existence of a flexible system that protects chromosomal integrity in whitefish and prevents changes in telomeric regions. This could serve as confirmation of the telo-hormesis hypothesis. A similar mechanism was observed in the Yenisei hump-nosed whitefish (*C. fluviatilis*), in which TL remained unchanged during temperature stress and 20 days thereafter, although changes in TA and the number of functionally active mitochondria were detected [58]. Interestingly, this mechanism is disrupted when the genome is destabilized by interspecific hybridization. In hybrids of Yenisei hump-nosed whitefish and Baikal whitefish, telomere shortening occurred under temperature stress [58].

It is important to note that the stress response is species-specific and highly dependent on the conditions under which the species has evolved, as well as the ontogenetic stage [16,83]. In embryos of *S. salar*, for example, TL increased under heat stress [27], while in adult *S. trutta*, increasing water temperature resulted in a shortening of telomeres, as was also observed in Siberian sturgeon (*Acipenser baerii)* [25]. Not only heat stress but also cold stress affects TL in fish, leading to its shortening [24]. The change in the optimal ambient temperature of the environment, whether upwards or downwards, acts as a stress factor for fish, leading to macromolecular damage in the cells through the overproduction of ROS, altering physiology and reducing survival [84,85,86].

### 4.2. Effect of Acclimation and Temperature Stress on the Expression of Genes Involved in Telomere Length Maintenance

During acclimation, the expression of the *trf2* gene is reduced in the larvae, while the activity of the other genes remains unchanged (Figure 3a and Figure 4). TRF2 regulates TL by stabilizing the t-loop and preventing telomere elongation [87]. The down-regulation of *trf2* expression in Baikal whitefish may facilitate telomerase function by allowing better access to telomeric DNA, which may be part of an adaptive mechanism.

Acute heat stress increases the expression of the *tzap* and *smg* genes in acclimated individuals, while it decreases the expression of *tert*, *ctc1*, *trir*, *rtel*, and *tpp1*. In non-acclimated individuals, stress reduces the expression of *atm*, *rtel*, and *tpp1* (Figure 3a and Figure 4). *Tzap* and *smg* are known to play a crucial role in the regulation of TL. TZAP is a protein that marks long telomeres and initiates trimming, the regulated shortening of excessively long telomeres [88]. Moreover, TZAP acts as a transcription factor for nuclear mitochondrial genes and is essential for mitochondrial metabolism by linking telomere and mitochondrial homeostasis [89]. The SMG family gene products contribute to the maintenance of telomere integrity by interacting with TERRA [90], regulating mRNA quality through nonsense-mediated mRNA decay (NMD) [91], and assisting telomerase interaction with single-stranded telomeric DNA during telomere elongation, particularly SMG6 [92]. The increased expression of *tzap* and *smg* likely ensures telomere maintenance and genome integrity. Additionally, TZAP regulates mitochondrial function, and this factor may remodel the transcriptional profile of antioxidant enzyme genes during acclimation.

The reduced expression of other genes may be associated with changes in the telomerase enzymatic activity. Under acute heat stress, both acclimated and non-acclimated larvae showed an increase in TA (Figure 2b), despite reduced expression of some key genes, including *tert* and *tpp1*, which are crucial for TL maintenance (Figure 3a and Figure 4). Notably, the increase in TA in acclimated individuals reached the control level, whereas TA in non-acclimated individuals exceeded the control level. This indicates remodulation of telomerase function during acclimation and an effective regulation of its activity through a feedback mechanism. In this mechanism, the expression of the *tert* catalytic subunit gene is reduced when TA increases to avoid negative effects. A similar response to seasonal temperature increases has been described in corals: while TA was not studied in corals, TL was maintained despite reduced expression of the *tert*, *trf2*, and *tpp1* genes [93]. In non-acclimated *C. baicalensis*, this feedback mechanism appears to be ineffective, as the cell continues to synthesize telomerase, and expression of the *tert* gene remains unchanged, leading to a greater expenditure of metabolic resources to cope with unfavorable conditions (Figure 4).

In addition to *tert*, the expression of *ctc1*, *rtel*, and *tpp1* was also reduced in acclimated individuals (Figure 3a and Figure 4). Normally, CTC1 stabilizes single-stranded telomeric DNA, making it inaccessible to telomerase. However, its knockout leads to pathological telomere elongation [94]. The reduced expression of *ctc1* may indicate the need to maintain telomeric regions and make single-stranded telomeric DNA available for telomerase, which has increased activity in non-acclimated individuals under stress (Figure 2b and Figure 4). TPP1 is required for the recruitment and stimulation of telomerase activity [95]. The reduced expression of *tpp1* in both non-acclimated and acclimated Baikal whitefish indicates their sensitivity to a sudden rise in temperature, as observed in corals [93]. However, in non-acclimated larvae, where *tert* expression is not reduced under stress (Figure 3a and Figure 4), the decrease in *tpp1* activity may indicate independent regulation of these genes and the involvement of other factors that assist telomerase in binding to single-stranded telomeric DNA. This may reflect an initial phase of cellular defense, as seen in the transcriptional profile of these individuals. In acclimated individuals, adaptation to temperature fluctuations occurs gradually over a long period of time, beginning early in ontogeny. In contrast, in non-acclimated individuals, this process requires more time, resources, and health, resulting in atypical gene activation or deactivation. Additionally, the reduction in the expression levels of the *atm* and *rtel1* genes indicates a weakening of telomeric DNA protection, allowing easier access for the telomerase complex. These genes typically become more active when telomeres shorten and less active when telomeres are longer.

Thus, the decreased activity of some telomeric genes and the increased activity of other genes in both acclimated and non-acclimated individuals could point to a regulatory mechanism for TL that frees telomeric DNA from proteins that negatively regulate TL, such as *atm* and *ctc1*, as well as from tertiary structures such as quadruplexes (*rtel* gene). This mechanism may enable more efficient telomere maintenance in acclimated individuals by linking telomere regulation to the overall cellular state (*tzap*, *smg*) and remodulating TA.

### 4.3. Different Activity Profiles of Antioxidant Enzyme Genes in Acclimated and Non-Acclimated Individuals

Almost any type of unfavorable stress to an organism results in activation of the antioxidant system, be it hypoxia [96], gamma radiation [97], toxic metal(loid) exposure [98,99], starvation [100], or temperature fluctuations [101]. This generally indicates that fish adopt a defensive (compensatory) strategy in response to stress [102]. In Baikal whitefish, changes in the expression of antioxidant enzyme genes were observed during both temperature acclimation and acute temperature stress. However, the expression profile varied depending on the type of exposure.

In acclimated individuals, a decrease in the gene expression of the antioxidant enzymes GPx3 and GST, which utilize the reducing potential of glutathione, was observed, while the expression of genes for superoxide dismutase and catalase remained unchanged (Figure 3b and Figure 4). The increased enzymatic activity of glutathione peroxidase GPx3 is typically found outside the cell and, in conjunction with the neutralization of hydrogen peroxide, may be involved in maintaining the bioavailability of nitric oxide in blood vessels, preventing the formation of blood clots, and suppressing tumors [103,104]. Glutathione S-transferases are a polymorphic family with a wide range of protective functions, including the neutralizing of electrophilic substances such as oxidized lipids, DNA, and various xenobiotics [105]. The decreased expression of the *gpx3* and *gst* genes in acclimated whitefish larvae compared to controls may indicate remodulation of their activity, similar to telomerase. It is likely that a shortage of reduced glutathione in the cell affects the expression of genes for glutathione-dependent enzymes. Normally, a decrease in glutathione levels signals severe oxidative stress [106], but the regulation of the ratio between reduced and oxidized glutathione is species-specific and depends on the intensity of the negative effects [107]. Not all salmonids respond positively to acclimation to warmer temperatures [108], further highlighting the species-specificity of responses to environmental change [109]. However, most species exhibit positive physiological changes during acclimation to higher temperatures, allowing them to conserve energy for growth and reproduction [62,63]. Assuming that acclimation does not have a negative effect on Baikal whitefish, the altered transcriptional profile, including reduced expression of *gpx3* and *gst*, may be a result of adaptation to temperature fluctuations. Interestingly, in some other fish species, acclimation had no effect on the glutathione-dependent antioxidant system [110], reinforcing its mild impact on the organism. Although we cannot completely rule out possible negative effects of acclimation, the acclimated larvae showed minimal changes in their transcriptional profile compared to controls and responded to acute temperature stress by restoring the gene expression levels of glutathione enzymes (Figure 4). This indicates that acclimation had a positive effect on Baikal whitefish.

Under heat stress, the gene expression of antioxidant enzymes is typically increased in fish. For example, the expression of superoxide dismutase and catalase genes increased with rising temperatures in different tissues of the cyprinid *Onychostoma macrolepis* [111]. The expression of the *sod*, *cat*, and *gpx* genes was elevated in the black porgy *Acanthopagrus schlegeli* [112]. In European sea bass *Dicentrarchus labrax*, the activity of the genes for the cytosolic forms of superoxide dismutase, but not that of catalase, increased when the fish were kept at 24 °C for two weeks [113]. However, when the fish were kept at 26 °C for one month, the activity of the catalase and glutathione peroxidase genes increased [114]. These data show that the degree and timing of exposure to high temperatures are crucial for the response to thermal stress: high sublethal temperatures and prolonged exposure lead to increased expression of all components of the antioxidant system, which can result in apoptosis and death. In acclimated *C. baicalensis* under acute temperature stress, an increase in the activity of cytosolic superoxide dismutase genes and their chaperone *ccs* was observed, while the activity of glutathione peroxidase genes was increased in non-acclimated individuals compared to the control group (Figure 4). Comparing the acclimated control individuals with acclimated individuals that were stressed, as described above, a recovery of the control level of glutathione enzyme gene expression (*gpx3* and *gst*), which was reduced after acclimation, is observed. The differences in expression levels become more apparent when comparing the groups of stressed acclimated and non-acclimated individuals: the expression of superoxide dismutase genes is lower in the non-acclimated individuals, whereas the expression of glutathione enzyme genes is higher (Figure 4). Superoxide dismutase is the first line of defense against ROS, and glutathione-dependent enzymes neutralize the resulting peroxides [115]. Consequently, acclimation adjusts the antioxidant system of fish so that the response to high temperatures becomes more efficient in the initial stages of stress due to the increased expression of superoxide dismutase genes (hormesis effect). In contrast, in non-acclimated individuals, oxidative stress spreads more rapidly within the cell, resulting in a secondary response characterized by increased expression of glutathione peroxidase genes. Moreover, this pattern may be a consequence of lower ROS formation in acclimated individuals, which also slows down the antioxidant response and is related to the hormesis effect.

## 5. Conclusions

The Baikal whitefish larvae acclimated during embryogenesis exhibit lower telomerase activity and altered gene expression profiles, indicating adaptation to temperature fluctuations compared to the control group. Acute temperature stress affects acclimated and non-acclimated individuals differently; however, telomere length does not change in all analyzed groups. Telomerase activity under stress returns to the control level in acclimatized individuals; the activity of the genes *tzap* and *smg*, whose products are involved in maintaining telomeres at a certain length, as well as that of the genes for the superoxide dismutase *sode_CuZn* and chaperone SOD ccs, increases. At the same time, the expression of several genes involved in the regulation of telomere length, including *tert*, is reduced. In non-acclimated individuals, telomerase activity increases, but the expression of several genes involved in telomere length maintenance decreases. Additionally, the glutathione peroxidase genes become more active. The difference in telomerase activity and transcriptional profiles between acclimated and non-acclimated larvae indicates better adaptation to sudden temperature changes in acclimated individuals. This is manifested by a remodulation of telomerase activity, the genes that regulate telomere length, and the antioxidant system, allowing acclimated larvae to cope better with stress conditions.

## Figures and Tables

**Table 1:** Names and functions of the gene products whose expression is analyzed in this work.

Name of the Gene	Description
Genes involved in the regulation of telomerase activity and telomere length	
*Tert*	Telomerase reverse transcriptase: catalytic subunit of telomerase; maintenance of telomeric DNA.
*Tep1*	Telomerase-associated protein 1: required for the amplification and localization of the telomerase complex.
*Ten1*	Telomeric pathways with STn1: protein binding to single-stranded telomeric DNA; involved in the negative regulation of the telomere length and capping, a component of the CST complex.
*Tpp1/Acd*	TINT1; PTOP; PIP1/Adrenocortical dysplasia phenotype: component of shelterin, bound to POT1 and TIN2, recruits telomerase and stimulates its processivity.
*Tcab1*	Telomerase Cajal’s body protein 1: part of the telomerase complex, bound to telomerase RNA, in S phase of the cell cycle assists in the amplification of the telomerase complex in Cajal’s bodies.
*Trf2*	Telomeric repeat-binding factor 2: a component of the Shelterin complex binding to double-stranded telomeric DNA; a negative regulator of the telomere length.
*Pot1*	Protection of telomeres protein 1: a component of the Shelterin complex, regulates the telomere length and telomerase activity at telomeres.
*Ctc1*	CST telomere replication complex component 1: regulation of the telomere length.
*Atm*	Ataxia-telangiectasia mutant phenotype: regulation of the telomere length and response to double-stranded DNA damage.
*Pin2/Trf1*	Protein involved in G2/M regulation/Telomeric repeat-binding factor 1: a component of the Shelterin complex, regulates the telomere length.
*Rif1*	Rap1p-interacting factor: negatively regulates the telomere length and is involved in DNA damage response, chromatin organization, and replication timing.
*Rtel1*	Regulator of the Telomere Length 1: helicase, involved in telomere maintenance and DNA reparation, is recruited to telomeres by the TRF1 protein to unravel G-quadruplexes, facilitating telomeric DNA replication.
*Tel2*	Telomere length regulation 2: involved in the response to DNA damage, stabilizes the TORC complex that regulates cell growth and survival.
*Trir*	Telomerase RNA Component Interacting Rnase: involved in the maturation of telomerase and other RNAs.
*Est1a/Smg6* *Smg5* *Smg7*	EST1 telomerase component homolog A/Suppressors with morphogenetic defects in genitalia proteins, effectors of nonsense-mediated messenger RNA decay: component of the telomerase complex, binding to single-stranded telomeric DNA, maintains the telomere length; has three isoforms: 5, 6, and 7.
*Sde2*	SDE2 telomere maintenance homolog: telomere silencing, genome stability, stress response, and cell cycle regulation.
*Tzap*	Telomeric zinc finger-associated protein: telomere trimming, prevents excessive telomere elongation.
Genes involved in the defense against ROS	
*Sodc_CuZn*	Copper, zinc superoxide dismutase cytosolic: converts superoxide radical to hydrogen peroxide in the cytoplasm.
*Sodm_FeMn*	Iron, manganese superoxide dismutase mitochondrial: converts superoxide radical to hydrogen peroxide in mitochondria.
*Ccs*	Copper chaperone for superoxide dismutase: activates cytoplasmic superoxide dismutase.
*Sode_CuZn*	Copper, zinc superoxide dismutase extracellular: reduces the amount of superoxide in the intercellular space.
*Cat*	Catalase: converts hydrogen peroxide into water.
*Gpx4* *Gpx3* *Gpx6*	Glutathione peroxidase: catalyzes the reduction in lipid hydroperoxides to the corresponding alcohols and the reduction of hydrogen peroxide to water.
*Gst_N* *Gst_C*	Glutathione S-transferase: has peroxidase activity, binds and neutralizes various ligands, including xenobiotics.

The gene description data were taken from the NCBI.

## Data Availability

The raw data supporting the conclusions of this article will be made available by the authors on request.
